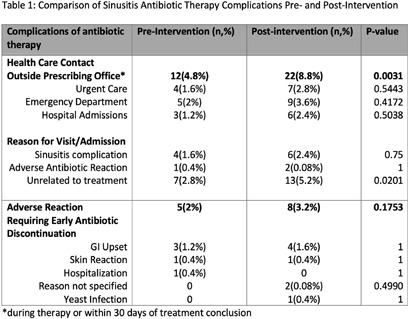# Assessment of a Prescription Feedback Intervention on Diagnosis, Management, and Safety Outcomes of Acute Bacterial Sinusitis

**DOI:** 10.1017/ash.2025.267

**Published:** 2025-09-24

**Authors:** Christine Kim, Michael Sportiello, Troy Anlage, Charles Springer, Monica Masterson, Colin Samoriski, Paulina Sudnik, John Bennett, Ankit Dahal, Lauraliz Delacruz, Jonathan Gigas, Jenny Kim, O, Alison Livada, Robert Fortuna, Jineane Venci, Alexandra (Sasha) Yamshchikov

**Affiliations:** 1 University of Rochester; 2 University of Rochester Medical Center; 3 University of Rochester Medical Center; 4 University of Rochester Medical Center; 5 URMC; 6 University of Rochester Medical Center; 7 URMC; 8 University of Rochester School of Medicine & Dentistry; 9 University of Rochester School of Medicine and Dentistry; 10 University of Rochester; 11 University of Rochester; 12 University of Rochester Medical Center; 13 university of rochester

## Abstract

**Introduction:** Although most rhinosinusitis cases are viral, misdiagnosis of an underlying bacterial cause is common, leading to excessive antibiotic utilization. Interventions to improve diagnosis and prescribing for sinusitis may reduce antimicrobial resistance and improve patient outcomes. **Methods:** Antibiotic prescriptions by 237 URMC Primary Care Network(PCN) clinicians between 9/1–11/31/2022 (baseline n=23,048) and 12/1/2023–2/29/2024 (post-intervention n=18,885) were extracted as part of a network-wide education and prescription feedback intervention focusing on antibiotic utilization rates, and guideline-concordant prescribing for sinusitis defined from local antibiograms and national guidelines. Random subsets of pre- and post-intervention prescriptions (250 each period for 90% power, Type I alpha=0.05) were systematically reviewed by two medical student or resident reviewers, with adjudication of discrepancies by infectious diseases-trained clinicians for 1) appropriate diagnosis of bacterial infection, 2) antibiotic selection, and 3) treatment duration. Charts were also reviewed for treatment failure requiring course extension, urgent care or emergency department utilization, hospital admission, and antibiotic-related toxicity during therapy or within 30 days of conclusion. Statistical analysis was performed in GraphPad and Excel ver. 2408. **Results:** Correct use of diagnostic criteria increased from 52.8% to 63.6% (p<0.01) post-intervention, a 10.8% absolute and 20.4% relative increase in appropriate diagnosis of bacterial sinusitis. Although rates of guideline-concordant treatment duration remained similar (74% vs. 74.4%, p=0.92), appropriate antibiotic selection increased from 71.6% to 85.6% (p< 0.01) (Fig. 1), and complete concordance of both spectrum and duration increased from 49.6% to 60.8% (p=0.012). Rates of antibiotic-associated adverse events requiring treatment discontinuation were similar pre- and post-intervention (Table 1). There were no Clostridium difficile infections. Health care contact beyond the prescribing office increased post-intervention (p < 0.01) (Table 1) but this was unrelated to sinusitis or antibiotic treatment complications (Fig. 2). Conclusions: A large-scale network intervention significantly improved use of diagnostic criteria and appropriate treatment of acute bacterial sinusitis without negatively impacting the incidence of treatment- or infection-related complications.

**Figure:**
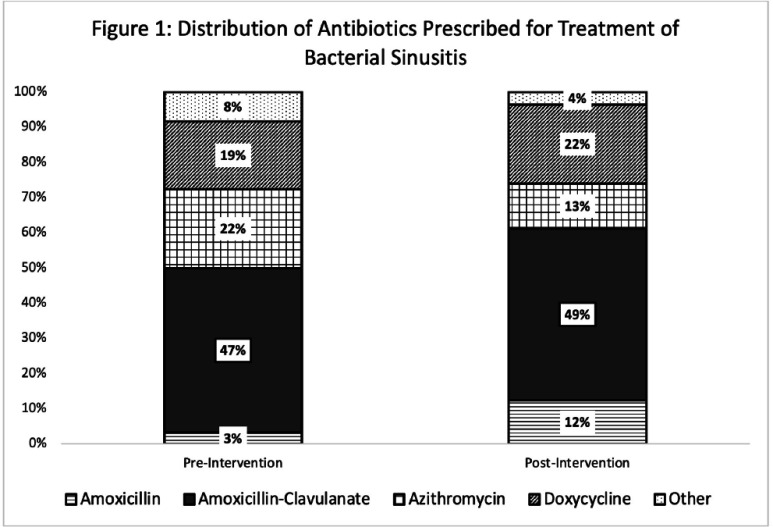

**Figure:**
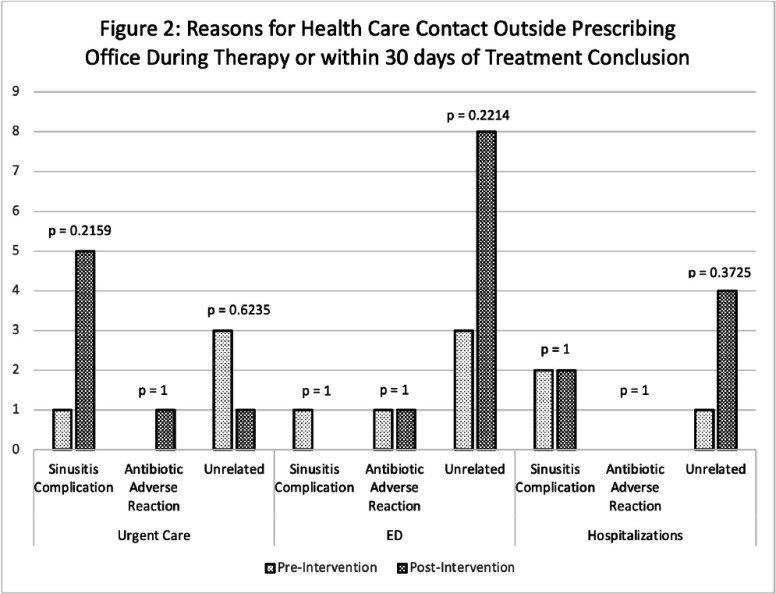

**Figure:**